# The possible association between neurodegenerative/demyelinating neurological disorders in achalasia patients

**DOI:** 10.1515/tnsci-2022-0269

**Published:** 2022-12-31

**Authors:** Salim T. Khoury, Amir Mari

**Affiliations:** Department of Neurology, The Nazareth Hospital EMMS, Ramat Gan, Israel, Nazareth, Israel; The Azreili Faculty of Medicine, Bar-Ilan University, Ramat Gan, Israel; Gastroenterology Institute, The Nazareth Hospital EMMS, Nazareth, Israel

## Abstract

The precise pathogenesis of achalasia is still unclear. Neurodegenerative and/or demyelinating disorders (NDD) appear to share some common pathophysiological pathways described in achalasia such as inflammation, autoimmune, mitochondrial dysfunction, and neurodegeneration. Jerie et al. have published on the October issue a prospective study assessing the prevalence of several NDD in achalasia patients. In this commentary, we shed some light on the possible link between achalasia and NDD as well as comment on the study by Jerie et al.

## 1 Introduction

Achalasia originates from the Greek word *a-khalasis*, meaning lack of relaxation. The precise pathogenesis of achalasia is still ambiguous. However, a theory of an autoimmune reaction provoked by a viral infection causing a cascade of an inflammatory process ending in destruction of the nitric oxide releasing neurons within the myenteric plexus has been widely proposed [1]. Achalasia is a chronic lifetime condition causing esophageal symptoms, malnutrition, and profoundly impacts patients’ quality of life [2]. Generally, the diagnosis of achalasia is delayed into some years. The classic endoscopic findings of achalasia present in about half of the cases and include dilated esophagus, food and fluid contents, and obstructed esophagogastric junction (EGJ). The modern gold standard modality for the diagnosis of achalasia is the high-resolution manometry (HRM) [3].

Neurodegenerative and/or demyelinating disorders (NDD) appear to share some common pathophysiological pathways described in achalasia such as inflammation, autoimmune, mitochondrial dysfunction, and neurodegenerative pathways [4]. However, despite the plausible common pathophysiological bases of achalasia and NDD, this link has never been properly addressed in clinical and epidemiological studies.

Jerie et al. have published on the October issue a prospective study assessing the prevalence of several NDD in achalasia patients [5]. The authors have assessed in a prospective comparative study the prevalence of both achalasia and NDD and compared them with their estimated prevalence in general population. Overall, 150 patients with achalasia and 112 patients with NDD were included. The authors have reported a higher prevalence of NDD among patients with achalasia (6.0%) as compared to the estimated 2.0% prevalence in general population (*p* = 0.003). On the contrary, the authors did not observe significantly increased prevalence of achalasia in patients with NDD compared to the general population. The authors have concluded that the prevalence of NDD was significantly higher among patients with achalasia compared to general population, suggesting an association of these disorders.

The hypothesis of this study and its findings are attractive and could further contribute to our understanding of achalasia.

Still, despite the interesting initial finding of this study, some comments arouse from it as follows:

We think that the sample size included in this study is quite small and further larger multicenter international studies should be considered to better address the prevalence/association between achalasia and NDD. Moreover, despite that 32 patients with NDD reported achalasia, only half of those agreed to undergo endoscopy and HRM to further diagnose/exclude achalasia. Therefore, some cases of achalasia could have been missed due to underdiagnosis.

Finally, the included NDD group of disorders is very heterogeneous and basically could have been included different disorders with various pathophysiological backgrounds (such as Parkinson’s disease, multiple sclerosis, epilepsy, and others).

One important factor upon considering neurodegenerative disorders is time. Presumably, looking at age subgroups and at the duration of disease could have revealed a clearer picture of a possible association between achalasia and NDD. With this in mind, symptoms, some of which may precede some of the neurodegenerative disorders by years, should have been included in the questionnaire; these include cognitive dysfunction, urinary disturbances, chronic constipation, REM-sleep behavioral disorder, and hyposmia.

Interestingly, the pathophysiology underlying dysphagia in Parkinson’s disease remains elusive. Impaired dopaminergic and non-dopaminergic mechanisms of the cortical swallowing network as well as brainstem and peripheral neuromuscular involvement have been suggested [6]. Recently, we have published a case of achalasia as diagnosed in HRM in a patient with Parkinson’s disease and dysphagia, probably as a sign of an inhibitory impairment [7]. The patient’s dysphagia has responded well to Levodopa and HRM normalized, a finding suggesting a brainstem origin of the problem rather than esophageal myenteric plexus dysfunction. This also suggests a dopaminergic modulation of the swallowing central pattern generator.

Putting all together, the results of this study are important and shed light on the association between achalasia and NDD. Still, future larger studies with more specific neurological diseases groups (probable multiple sclerosis) are warranted to further better address this plausible relationship.

## Abbreviations

EGJ: esophagogastric junction
HRM: high-resolution manometry
NDD: neurodegenerative and/or demyelinating disorders
